# Editorial: The Use of Phytogenic Feed Additives to Enhance Productivity and Health in Ruminants

**DOI:** 10.3389/fvets.2021.685262

**Published:** 2021-05-04

**Authors:** Ahmed E. Kholif, Uchenna Y. Anele, Amlan Kumar Patra, Zora Varadyova

**Affiliations:** ^1^Dairy Science Department, National Research Centre, Giza, Egypt; ^2^North Carolina Agricultural and Technical State University, Greensboro, NC, United States; ^3^Department of Animal Nutrition, West Bengal University of Animal & Fishery Sciences, Kolkata, India; ^4^Centre of Biosciences, Institute of Animal Physiology, Slovak Academy of Sciences, Kosice, Slovakia

**Keywords:** plant secondary metabolites, ruminants, phytogenic feed additives, animal health, nutraceutical

Plant secondary metabolites (PSM) are biologically active compounds which can exert beneficial effects on ruminal fermentation, feed digestion, health, and productivity (1, 2). Due to their antimicrobial effects against undesirable ruminal bacteria, protozoa, and methanogens, PSM can serve as alternatives to antibiotic feed additives in ruminant production (1, 2). The special issue (12 articles: 9 research papers and 3 review articles) provides recent insights on new phytochemicals with potent activities, mechanisms of action, their synergistic effects, and interactions with other compounds.

Three reviews were published in this special issue. The review article by Hassan et al. discussed the ability of phytogenic additives to modulate rumen microbiome, fermentation kinetics and methanogenesis mediated through diet–microbe-phytochemical interactions. Authors concluded that pure PSM, plant extracts or PSM-rich phytogenic feeds have the ability to modulate rumen microbiota, increase volatile fatty acids and decrease ammonia and methane (CH_4_) production. Inhibition of enteric CH_4_ emission was consistently observed in *in vitro* experiments, while the *in vivo* effects varied greatly. The authors emphasized that many factors contribute to this vast variability, including variations of the chemical compositions and dose of the PSM, application methods, dietary composition, physiological stage of animals, feeding conditions, and progressive adaptation of microbes for specific phytochemicals. Moreover, they discussed the effects of production, extraction, processing, and application of phytochemicals on the expected responses. In another review, Sun summarized the effects of glucosinolates present in brassica forages on CH_4_ emissions from ruminants, and concluded that brassica forages can be used as a useful tool to reduce CH_4_ emitted per unit of DM intake compared with grass-based forages. The author explained physiological mechanisms in the mitigation of CH_4_ emissions from ruminants fed brassica forages beyond the direct inhibitory effect on methanogens or fermentation profile changes. Glucosinolates and/or their ruminal microbial breakdown products are absorbed into the blood and then may stimulate the secretion of thyroid hormone causing many changes in rumen physiology including a reduction in ruminal digesta retention time, thereby reducing CH_4_ emissions In the third review article, Tedeschi et al. reviewed the ecologically relevant phytochemicals including polyphenolics, terpenes antioxidants, alkaloids, flavonoids, condensed tannins and essential oils on ruminant performance, and their sustainable production and utilization to replace antibiotics. They recommended the use of many phytochemicals at the same time to obtain better biological responses in ruminant production.

The effect of phytogenic feed additive supplementation on *in vitro* fermentation was evaluated in 3 research articles. In one experiment, Ebeid et al. noted that *Camelina sativa* oil (0, 2, 4, 6, and 8% of dry matter) changed proportions of individual ruminal volatile fatty acids and decreased CH_4_ production by altering total bacteria, protozoa, and methanogens populations. *Camelina sativa* oil caused a linear decrease in bacterial richness and evenness indices along with Shannon diversity. In another experiment, Ahmed et al. reported that Mootral [a commercial product containing *Allium sativum (*garlic) powder and *Citrus aurantium* (bitter orange) extracts] at 0, 10, and 20% of the substrate altered ruminal microbial community, produced more propionate, and reduce microbial groups associated with CH_4_ production *in vitro*. Such effects were reflected as reducing CH_4_ percentage and CH_4_/CO_2_ ratio in a dose-dependent manner. Moreover, Mootral increased abundances of H_2_-consuming groups such as *Prevotellaceae* and *Veillonellaceae* and reduced some H_2_-producing bacteria as well as reduced the major CH_4_-producing family *Methanobacteriaceae* and increased *Methanomassiliicoccaceae*. Gomaa et al. reported that red osier dogwood extract at 1% of DM in the rumen simulated technique system tended to decrease acetate to propionate ratio and decreased starch disappearance in the barely-based diet, but not in the diet containing dried distillers grains with solubles. Rumen microbiota was not affected by red osier dogwood extract at the phylum level, but was altered at the genus level.

The response of ruminants to phytochemicals *in vivo* was evaluated in 6 studies. Hassan et al. observed that the supplementation of Murrah water buffaloes with a mixed phytogenics (ginger tuber, turmeric tuber, licorice roots, fennel seeds, fenugreek seeds, ajwain seeds, *Swertia chirata* leaves, *Terminalia chebula* fruit, *Citrullus colocynthis* fruit, and *Phyllanthus emblica* fruit in equal quantities) at 0, 15, 25, and 35 g/d for 6 weeks resulted in substantial changes in the rumen bacteriome composition (increased the abundances of *Firmicutes* and *Proteobacteria* phyla, and decreased the abundance of *Bacteroidetes* phylum and *Prevotella* genus) and milk fatty acid (increased *n*-3 fatty acid content and decreased stearic acid content). Heat stress in livestock causes reduction in production performance, immune responses, and health status as well as imbalances of stress-related hormones due to decreased antioxidant status, which can be ameliorated by supplementation of herbs (3). The study of Li et al. also showed that supplementation of mulberry leaf flavonoids (0, 15, 30, and 45 g/d) for 5 weeks, decreased the oxidative stress marker (e.g., malondialdehyde concentrations) and total antioxidant capacity and catalase while increasing the serum heat shock proteins, glutathione peroxidase, and insulin concentrations in Murrah buffaloes. The highest dose of mulberry leaf flavonoids at 45 g/d was the most appropriate dose for supplementation in lactating buffaloes to enhance lactation performance and alleviate heat-induced oxidative stress during the summer season. Additionally, Li et al. evaluated the effects of *Radix puerarin* extract supplementation for 60 days at 0, 200, 400, and 800 mg/kg in the feed concentrate and reported that *R. puerarin* extract at 400 mg/kg improved growth performance and meat quality of heat-stressed beef cattle by improving muscle total antioxidant status, superoxide dismutase, and glutathione peroxidase activity and reducing muscle fiber diameter, shear force and myosin heavy-chain (fast-glycolytic) muscle fiber composition. Galván et al. included a polyherbal feed mixture (*Achyrantes aspera, Trachyspermum ammi, Citrullus colocynthis, Andrographis paniculata*, and *Azadirachta indica*) in diets of growing calves at 0, 3, 4, and 5 g/d and noted that additives at 4 g/d showed the best performance results with improved growth and health status during the pre-ruminant to the weaning period through modification of different gene expression, notably biological processes associated with tight junction, mucin biosynthesis, and intestinal immunoglobulin A production. Moreover, they reported that polyherbal treatment could improve the metabolism of lipids, carbohydrates, proteins, and also immune response revealed through gene expression analysis. Petrič et al. evaluated the effect of a mixture of dry medicinal herbs (*Fumaria officinalis* L., *Malva sylvestris* L., *Artemisia absinthium* L., and *Matricaria chamomilla* L.) at 100 g DM/d and organic zinc at 70 mg Zn/kg diet on ruminal microbial fermentation and histopathology in lambs for 70 d. Supplements did not affect the ruminal fermentation parameters or the protozoal population of the lambs; however, they lowered total ruminal bacterial population. Additionally, the study showed that long-term dietary supplementation with organic zinc combined with a mixture of medicinal herbs with a strong antioxidant capacity could negatively affect the health of the ruminal epithelium. The findings highlight that the effect of dry medicinal herb mixtures depends on the variety and synergy of herbal polyphenols and the combination of bioactive compounds. Jiao et al. evaluated the effect of feeding cobalt and oregano essential oil blend in ram diets and reported improved cellulose and nutrient digestibility.

In summary, the research within this special issue, along with many other publications elsewhere, demonstrated the ability of phytogenic feed additives to modify nutrient digestion and ruminal microbes as well as improve health and host metabolism resulting in enhanced ruminant performance and abatement of CH_4_ production. The effect of phytochemicals is dose-dependent revealing the importance of defining the optimal dose of each phytochemicals under specific conditions.

## Author Contributions

AK, UA, AP, and ZV have served as editors of the Research Topic and have co-written the editorial. All authors contributed to the article and approved the submitted version.

## Conflict of Interest

The authors declare that the research was conducted in the absence of any commercial or financial relationships that could be construed as a potential conflict of interest.
